# A prospective multi-site study to evaluate the performance and usability of an oral fluid-based HIV self-test in Canada

**DOI:** 10.1186/s12889-024-21228-8

**Published:** 2025-01-11

**Authors:** Richard A. Galli, Darshanand Maraj, Kristin McBain, Jason M. Lo Hog Tian, Abbey McFarland, Wangari Tharao, Nontobeko P. Nkala, Arlene Chan, Mark da Silva, Rejean Thomas, Anne-Fanny Vassal, Martin Lepage, Laurie Ireland, Mike Payne, Jared Starr, Chris Fraser, Marion Selfridge, Mona Loutfy, Roberta Halpenny, Nirubini Jeyarajah, Vanessa Tran, Tony Mazzulli, Sean B. Rourke

**Affiliations:** 1 MAP Centre for Urban Health Solutions, Unity Health Toronto, Toronto, ON Canada; 2 University of Toronto, Toronto, ON Canada; 3 Women’s Health in Women’s Hands Community Health Centre, Toronto, ON Canada; 4 Hassle Free Clinic, Toronto, ON Canada; 5 Clinique Médicale L’Actuel, Montreal, QC Canada; 6 Nine Circles Community Health Centre, Winnipeg, MB Canada; 7 Department of Family Medicine, University of Manitoba Rady Faculty of Health Sciences, Winnipeg, MB Canada; 8 Cool Aid Community Health Centre, Victoria, BC Canada; 9 Maple Leaf Research, Toronto, ON Canada; 10 Women’s College Hospital, Toronto, ON Canada; 11 Public Health Ontario Laboratory, Toronto, ON Canada; 12 Department of Laboratory Medicine and Pathobiology, University of Toronto, Toronto, ON Canada

**Keywords:** HIV testing, OraQuick®, Oral fluid-based HIV self-test, Accuracy, Usability, Acceptance

## Abstract

**Background:**

Blood and oral fluid-based HIV self-tests are important for reaching the undiagnosed living with HIV. The study objectives were to evaluate the oral fluid-based OraQuick® HIV Self-Test (HIV-ST) performance in comparison to laboratory reference testing; determine if laypersons can correctly perform the HIV-ST; document if intended users can successfully interpret pre-made contrived positive, negative, and invalid results; and document if intended users can understand the key messages in the product labeling.

**Methods:**

This prospective study enrolled consenting adult intended users of HIV self-testing from six community health centres in four Canadian provinces between June 2022 and January 2024. Positive and negative agreement was determined by comparing the results of the HIV self-tests with the results of the laboratory-based “gold standard” Abbott Alinity HIV Antigen/Antibody Combo test. Descriptive statistics were used to summarize usability self-test procedure steps.

**Results:**

Overall, 951 participants were recruited and consented with 911 available for all analyses. With respect to sociodemographics: 84% of participants were between 18–45 years of age, 73% had at least a college education, 48% were Cis-male, 45% were employed; and 26% identified as White, 23% as African, Caribbean or Black, 5% as Indigenous [First Nations, Métis or Inuit], 33% as Asian, and 6% as LatinX. Primary efficacy analysis on the 911 who completed HIV-ST revealed a single confirmed positive participant and a negative percent agreement of 100% (880/880, 95% CI: 99.9–100%) with the comparator method. For usability determination, the average success rate for “critical” steps for completing the test was 94.1%. Approximately 97% of participants found the instructions easy to follow and 98% of participants reported they would use the test again. Of the 465 participants who interpreted the strong positive, weak positive, negative, and invalid pre-made contrived results, the average of correct interpretations ranged from 59–97%

**Conclusions:**

A licensed oral fluid-based HIV self-test in Canada can present an accurate, easy-to-use, and less invasive alternative to blood-based HIV testing. The addition of an oral-fluid self-test along with the current licensed blood-based HIV self-test could help reach the undiagnosed with HIV in Canada and positively impact HIV testing rates overall by offering individuals a choice of self-testing devices.

**Supplementary Information:**

The online version contains supplementary material available at 10.1186/s12889-024-21228-8.

## Background

Across Canada, HIV diagnosis rates continue to rise. There were 2,434 new HIV diagnoses in 2023 (35.2% increase since 2022) with the national rate of new HIV diagnoses rising to 6.1 per 100,000 population in 2023 compared to 4.7 per 100,000 population in 2022. The highest rates were reported in the prairie provinces of Saskatchewan and Manitoba [1]. Although Canada has endorsed the Undetectable = Untransmittable (U = U) declaration and the UNAIDS 95–95-95 targets (95% diagnosed, 95% of those on treatment and in care, and 95% of those achieving viral suppression by 2030), much still needs to be done to reach those targets [2]. From the most recent published data as of the end of 2022, an estimated 65,270 people were living with HIV in Canada with an estimated 89% diagnosed. Of those diagnosed, 85% were estimated to be on treatment with 95% of those having a suppressed viral load [3]. Collectively, as of the end of 2022, there were still nearly 16,000 people who are undiagnosed, or who are diagnosed but not yet on antiretroviral therapy in Canada and therefore not engaged in the continuum of HIV care. These gaps in diagnosis and connection to care and treatment are drivers of the epidemic in Canada and need to be addressed.

The acceptance and usability of oral fluid and blood-based HIV self-tests by key populations in global settings have been well established [4–6]. In Canada, recent novel testing approaches using finger stick blood-based HIV self-testing to reach the undiagnosed, such as I’m Ready and GetaKit, have shown high uptake, particularly amongst key and underserved populations such as gay and bisexual men who have sex with men, African, Caribbean, and Black populations, people who use substances, women, youth and Indigenous peoples. Self-testing programs can also provide information and “do-it-yourself” pathways for support, care, and treatment management, and have been shown to be broadly acceptable and effective in reaching the undiagnosed in Canada and linking them to care [7–9]. Furthermore, studies of HIV self-test implementation in Ontario, Canada found that programs providing low-barrier access to self-testing effectively reached key and underserved populations, identified persons living with undiagnosed HIV, and linked them to care and treatment [10–12]. A fundamental step towards ending the HIV epidemic by 2030 is reaching the undiagnosed which remains a critically important first step for people to know their status and get engaged and connected to treatment, care and prevention as appropriate [13, 14].

Following the World Health Organization (WHO) recommendation in 2016 for the use of HIV self-testing as an alternative to conventional facility-based testing [15], the WHO reported in 2024 on a wide array of countries adopting policies that include having both blood or oral fluid-based HIV self-testing as a vital part of differentiated HIV testing approaches [16]. This followed the UNITAID report in 2018 on the importance of the then-nascent HIV self-test development for use by key and underserved populations who may not choose to access facility-based testing alternatives [17].

In reviews published by Pai and colleagues on HIV self-test use, including oral fluid-based self-tests, in populations with high and low risk of acquiring HIV it was shown that HIV self-testing was broadly acceptable, and also that implementation of technology tools such as a mobile phone application can help increase testing uptake and facilitate linkages to care [18, 19]. In qualitative surveys of Canadian healthcare and community-based service providers, respondents reported that HIV self-testing is desired by Canadians and organizations were ready to support HIV self-testing. Yet, gaps remain in self-test accessibility and linkage-to-care pathways that need to be addressed in order for HIV self-testing programs to be fully established [20] (Penny L, Lo Hog Tian J, McBain K, Galli R, Gahagan J, Worthington C, et al: HIV Self-testing and Mobile Health: A National Survey of Community-Based HIV Service Providers in Canada, submitted).

The first blood-based HIV self-test was approved for use in Canada by Health Canada on November 3, 2020 [21, 22], with uptake and scale-up of self-testing continuing across the country through targeted intervention programs [10–12, 23]. In a scoping review of global studies based on the actual use of blood and oral fluid-based HIV self-testing, covering a period from 2011–2022, Adepoju et. al found a preference for oral fluid HIV self-testing over blood-based self-testing [24]. Since Canada currently has only a single, blood-based HIV self-test approved for use, the over-arching goal of this study was to provide performance outcome data on the OraQuick® HIV Self-Test (OraSure Technologies Inc., Bethlehem, Pennsylvania, USA) that are necessary for regulatory approval of an oral fluid-based HIV self-test in Canada which would provide self-testers with a choice of self-test devices to best meet their preferences for testing.

The specific study objectives were to evaluate the device performance, i.e., positive and negative percent agreement with laboratory reference testing (4th generation HIV antigen/antibody test); document if intended users (i.e., laypersons) can successfully perform the procedural steps of the OraQuick® HIV Self-Test without any training or demonstration of the test (accuracy/usability); document if intended users can correctly interpret pre-made contrived strong positive, weak positive, negative, and a range of invalid results in addition to their own results; and document if intended users can understand the key messages in the labelling (label comprehension).

The design and objectives of this study are aligned with the necessary Health Canada requirements for the evaluation of the performance and effectiveness of the OraQuick® HIV Self-Test. The *Health Canada Guidance for Manufacturers of Human Immunodeficiency Virus (HIV) Rapid Diagnostic Tests (RDTs) for use at the Point of Care (POC) or for Self-Testing* provides performance targets that must be met for clinical sensitivity and specificity: “Evidence that the RDT intended for use at the POC or for self-testing has a minimum sensitivity and specificity of ≥ 99% for HIV antibody detection should be provided” [25].

## Methods

### Study design

This prospective study enrolled consenting adults from six community health centres and medical clinics across Ontario, Québec, Manitoba, and British Columbia between June 2022 and January 2024.

### Inclusion criteria

The study was open to the general public, either English or French-speaking, aged 18 or older who met the inclusion criteria for enrolment and did not meet any of the exclusion criteria listed in *Appendix A.* Although individuals under 18 years of age were not eligible for enrolment, no participant was excluded based on any other sociodemographic characteristic. A participant compensation of $50 CAD was provided for their initial study site enrollment visit and an additional $25 CAD was provided on their second visit when they returned to receive their laboratory testing results.

### Study population and sample size

Recruitment of participants into the study occurred directly at the study sites during routine clinic visits using brochures, posters, and word of mouth. Participant’s self-reported HIV risk activity from the previous 12 months was collected at the time of study enrolment. The target sample size for the study was a minimum of 900 individuals with unknown HIV status including at least 400 individuals determined to be at risk of HIV infection, as specified by Health Canada for HIV self-test prospective studies. Because this study was designed to meet regulatory requirements for the number of participants based on the published guidelines [25], no sample size calculations were conducted.

### Study protocol

Upon obtaining informed consent from each participant, one venous blood sample was collected for testing at respective provincial central laboratories by the Comparator Method (CM). Each participant then undertook the self-test process at the study site which required use of the test stick device to self-swab the gums for oral fluid collection onto an integrated absorbent pad. Participants then performed the OraQuick® HIV Self-Test on the collected fluid and interpreted the results using only the manufacturer’s instructions for use as a guide. All steps in the self-test performance were directly observed by a study Observer who was a trained healthcare provider at the study site and who did not instruct, demonstrate the test, intervene or otherwise interact in any way with the participant conducting the OraQuick® HIV Self-Test. Observations about the participant’s test experiences were noted throughout the self-testing process. The results of the rapid, lateral flow OraQuick® HIV Self-Test performed and interpreted by the study participants were recorded as Non-Reactive, Reactive or Invalid and were subsequently compared with the results of a licensed 4th generation, chemiluminescent microparticle immunoassay (CMIA)-based HIV-1/HIV-2 combination antigen/antibody (Ag/Ab) test CM (Abbott Alinity HIV Ag/Ab Combo test, Abbott Laboratories, Abbott Park, Illinois, USA) [26] that is in routine use at the central public health laboratories of each of the participating provinces. Alinity HIV Ag/Ab Combo test results are reported as Non-Reactive (considered negative for HIV-1 p24 antigen and antibodies to HIV-1 and HIV-2) or Reactive (to undergo supplemental confirmatory testing). The Geenius™ HIV 1/2 Confirmatory Assay (Bio-Rad Laboratories, Marnes-la-Coquette, France), which is an immunochromatographic test for the detection and differentiation of individual antibodies to HIV-1 and HIV-2, was used for positive confirmation where necessary. Results are interpreted as Positive, Negative, or Indeterminate for HIV antibodies based on criteria indicated in the package insert [27].

For usability determinations, there were 18 items listed in the survey which were similar to those utilized in an evaluation of several blood and oral fluid-based HIV self-test devices in South Africa [28]. These survey items were designed to capture information on participant’s use of the test kit instructions, preparations for the self-test, self-collection of the oral fluid sample, completion of the self-test procedure, and related post-test procedures. Of these 18 items, 10 were considered “critical” for correct usability (identified with * in Table 5). Immediately after the participant completed the self-test, they indicated their result interpretation to the Observer, who also interpreted the HIV self-test result and recorded the result independently. In addition, to comply with Health Canada requirements, a minimum of 400 volunteer participants were provided with blind-coded pre-made contrived result devices in random order and asked to interpret the test results.

The CM testing on serum from venous blood samples collected from study participants was conducted by the provincial public health laboratories and the results were provided back to the sites. Results were also uploaded to the central database at St. Michael’s Hospital, Toronto. The central laboratory test results were used as the reference result for the primary efficacy evaluation of the OraQuick® HIV Self-Test. To allow for adequate turnaround time for laboratory results to be provided back to the clinic, study participants were advised to return to the clinic approximately two weeks after their initial visit to receive their laboratory testing results. All HIV-related clinical care and prevention services provided to each participant were based only on the results of the standard HIV testing that is in normal use at each site, and not on the self-test result.

### Electronic data capture

Participant data were entered directly by the study Observers into tablets pre-loaded with study-specific surveys developed in Qualtrics (Qualtrics, Provo, UT, USA; https://www.qualtrics.com). This collected data was saved and stored on local secure and encrypted servers in Toronto at St. Michael’s Hospital for further data analysis. Only designated research team members were provided access to the password-protected data from the Qualtrics servers. To maintain privacy and confidentiality, participant survey data was linked only with their unique, anonymized participant ID. The full survey instruments that were developed for this study are provided in *Appendix C*.

### Ethics approval

This study was approved by respective Research Ethics Boards (REB) for the Ontario, Québec, Manitoba, and British Columbia sites participating in the study: St. Michael’s Hospital REB, University of Toronto REB, Veritas IRB Québec and British Columbia, and University of Manitoba HREB. This study was conducted under a Health Canada-approved Investigational Testing Authorization (ITA), application ITA# 336,702, issued on 11 May 2022 and is listed in ClinicalTrials.gov ID# NCT05020353**.**

### Data analysis

The study data analysis was conducted using IBM SPSS Statistics version 24. Sociodemographic variables and usability characteristics were summarized using descriptive statistics. The device performance (Primary Efficacy) was analyzed through the calculation of positive and negative percent agreement between study participants’ self-interpreted results of the OraQuick® HIV Self-Test versus the 4th generation Abbott Alinity test results received from the provincial laboratories. For data analysis purposes, each study participant’s “true” HIV status was determined upon completion of the CM and confirmatory testing if necessary, and the participant’s interpretation of their self-test result then determined whether their self-test results were included in either the positive percent agreement (PPA) population or the negative percent agreement (NPA) population. All invalid self-test results, those with “inconclusive” laboratory results and responses indicated as “do not know” were excluded from the calculation of PPA and NPA.

For PPA and NPA calculations, the overall 95% confidence interval was determined where:

- PPA = [TP / (TP + FN)] × 100, where TP (true positive) is positive self-test in agreement with positive Alinity test, and FN (false negative) is negative self-test discordant with positive Alinity test; and,
- NPA = [TN / (TN + FP)] × 100, where TN (true negative) is negative self-test in agreement with negative Alinity test, and FP (false positive) is positive self-test discordant with negative Alinity test.
  In addition, the study participants’ self-interpreted results were compared with the Observers’ interpretation of the self-test results.

## Results

### Study participants

**Fig. 1:**
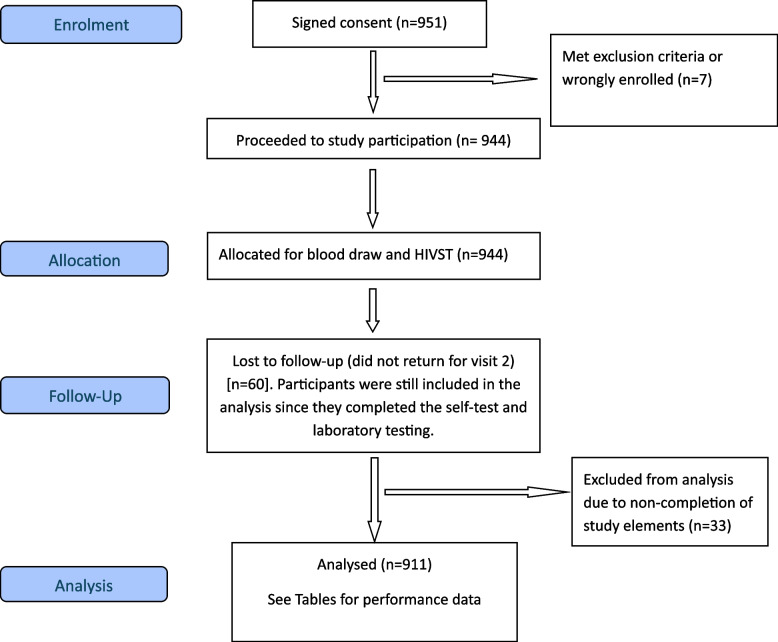
Participant enrolment CONSORT flow diagram

A total of 951 participants were enrolled over the study period. Of these, 40 participants were excluded from all analyses for reasons including failure to provide a venous blood sample or failure to provide a self-test result (see *Appendix B* for details on excluded participants). Figure 1 illustrates the CONSORT diagram for the number of participants who proceeded to enrolment, allocation, follow-up, and analysis within the study.

Table 1 presents the sociodemographic profile of the 911 participants who were included in the primary efficacy analysis. Overall, the sample recruited across all sites had the following sociodemographic characteristics: 84% of the sample was between the ages of 18 and 45 years of age; 50% identified as “Cis-women”, 48% identified as “Cis-men” and 3% identified as other (11 identified as “Non-binary”, 7 indicated Trans woman, 4 indicated “Genderqueer” i.e. gender identity that is fluid, changing, 2 indicated Trans man and 2 who self-described gender). There were also 7 participants who indicated that they were “Two Spirit” (a person who embodies both a masculine and feminine spirit) and 5 participants who indicated that they were pregnant. Regarding ethno racial composition, of the 911 who provided survey responses, approximately 33% (*n*=307) identified as “Asian” descent, 26% (*n*=235) identified as “White”, 23% (*n*=211) as “African/Caribbean/Black”, 5% (*n*=46) as “Indigenous”, 6% (*n*=54) as “LatinX”, 3% (*n*=25) as “Middle East/North African” and the remaining 4% (*n*=33) as “other”. Of the 908 participants who provided their educational achievement, approximately 46% (*n*=420) had a university degree or higher, 27% (*n*=242) completed College, 25% (*n*=226) completed High School, and 2% (*n*=20) completed primary school. Only 4% (*n*=39) of the 905 participants who responded reported having any kind of reading or writing impairment, and 33% (*n*=301) indicated visual impairment. Of the 904 participants who responded to the question on employment, 45% (*n*=405) were employed, 29% (*n*=261) indicated that they were students, 24% (*n*=215) were unemployed, and 3% (*n*=23) were retired.

**Table 1:** Participant demographics (*n* = 911)

	n	%
Age groups (*n* = 910)		
18–25	307	34%
26–35	302	33%
36–45	158	17%
46–55	73	8%
> 55	70	8%
Gender (*n* = 910)		
Cis-male	434	48%
Cis-female	450	50%
Other	26	3%
Ethnicity (*n* = 911)		
White	235	26%
African/Caribbean/Black	211	23%
Indigenous	46	5%
Middle Eastern/North African	25	3%
East Asian	85	9%
South Asian	222	24%
LatinX	54	6%
Other	33	4%
Sexual Orientation (*n* = 890)		
Heterosexual	606	68%
Gay/Lesbian	150	17%
Bisexual	82	9%
Other	52	6%
Education (*n* = 908)		
No formal education	0	0%
Primary	20	2%
Secondary	226	25%
College	242	27%
University or higher	420	46%
Employment (*n* = 904)		
Employed	405	45%
Student	261	29%
Retired	23	3%
Unemployed	215	24%
Medical Condition (*n* = 143)		
Diabetes	21	15%
Hypertension	23	16%
Sexually transmitted infection	13	9%
Other medical condition	86	60%
Reading/Writing Impairment (*n* = 905)		
No	866	96%
Yes	39	4%
Visual Impairment (*n* = 905)		
No	604	67%
Yes	301	33%
Tested for HIV Before (*n* = 901)		
No	422	47%
Yes	479	53%
HIV Status (*n* = 911)		
Negative status	485	53%
Unknown status/never tested	426	47%
Self-Reported Risk^a^ (*n* = 911)		
High Risk	520	57%
Low Risk	391	43%

^a^“High Risk” contains participants who self-reported one or more risk category

A total of 143 participants self-reported medical conditions, including hypertension (16%), diabetes (15%); and recent or existing sexually transmitted diseases (9%); approximately 60% disclosed “other” medical conditions (e.g., mental illness, pre-diabetes, Hepatitis C, asthma, and cancer were the most common). Regarding the 911 who self-reported HIV status, 47% (*n*=426) indicated that their status was unknown or had never been tested, while 53% (*n*=485) were negative indicating prior experience with HIV testing.

When evaluating the self-reported HIV risk category, 57% of the 911 participants (*n* = 520) reported at least one behaviour characteristic that would be considered “high risk”. In terms of specific self-reported high-risk characteristics: 31% identified as having condomless sex with men, while 21% had unprotected sex with women. Approximately 19% indicated having multiple sexual partners, 44 participants (5%) indicated having used injection drugs, 17 participants (2%) self-identified as sex workers, and 9 individuals (1%) indicated that their sexual partner was HIV positive.

### Primary efficacy (Performance) analysis

The primary efficacy analysis on the 911 who completed the OraQuick® HIV Self-Test showed a positive percent agreement of 100% (1/1) and a negative percent agreement of 100% (880/880, 95% CI: 99.9–100%) when comparing participant’s self-test results to the Abbott Alinity (see Table 2 for a full comparison of participant and laboratory test results and Table 3 for PPA and NPA calculations). The study encountered one previously undiagnosed participant living with HIV that was confirmed by the Abbott Alinity and Geenius™ comparator methods. The overall percent agreement between participant and observer for the self-test results was 97.6% (884/906) (see Table 4 for a full comparison of participant and observer interpretations of HIV-ST results). The overall “invalid” rate for the HIV Self-Test was 2.2% (*n* = 20) and an additional 1.0% (*n* = 9) of participants indicated they were uncertain (“do not know” or “unsure”) as to how to interpret the results.

**Table 2:** Self-Test Interpretation by the Participant Compared with Lab Result

			**ARCHITECT/Alinity Lab Result**	
**OraQuick Self-Test Result**	Negative	Positive	Inconclusive	**Total**
Negative	880	0	1	881
Positive	0	1	0	1
Don't know/Not sure	9	0	0	9
Invalid/Test did not work	20	0	0	20
**Total**	909	1	1	**911**

A total of 911 participants completed the OraQuick® self-test and ARCHITECT/Alinity laboratory testing. The OraQuick® Self-Test results listed in the first column are distributed across each row according to the corresponding laboratory (Architect/Alinity) comparator method results

**Table 3:** Positive and Negative Percent Agreement with Laboratory Results based on 911 completed HIV Self-Tests

True Positive	1
False Negative	0
True Negative	880
False Positive	0
Inconclusive^a^	1
Positive % Agreement	**100%** [1/1]
Negative % Agreement^b^	**100%** [880/880; 95% CI: 99.9–100]

^a^1 participant whose HIV status was inconclusive based on the results of laboratory testing was not included in the calculations

^b^A total of 29 invalid (*n* = 20) or do not know/not sure (*n* = 9) results were excluded from the calculation of negative percent agreement

**Table 4:** Participant and observer interpretations

	**Observer Interpretation**				
**Participant Interpretation**	Negative	Positive	Don't know/ Not sure	Invalid/Test did not work	**Total**
Negative	864	4	4	5	877
Positive	0	1	0	0	1
Don't know/Not sure	6	1	2	0	9
Invalid/Test did not work	1	1	0	17	19
**Total**	871	7	6	22	**906**^**a**^

A total of 906 participant-interpreted results were also interpreted by the study observers. The Participant-interpreted self-test results listed in the first column are distributed across each row according to the corresponding Observer interpretation

^a^5/911 observer results interpretations were not uploaded into the database

### Usability assessment

A total of 911 participants were included in the usability assessment surveys conducted by the study Observers. Across the 18 usability assessment questions in the survey, the average for all expected outcomes was 94.1%. For the 10 items identified as critical, the average of expected outcomes was also 94.1% (see Table 5 for a full breakdown of the Observers’ usability assessment). 83.2% of participants were able to correctly collect the oral-fluid sample, 5% were observed to incorrectly touch the absorbent pad during the collection procedure and 98% were able to correctly place the test device into the developer vial after sample collection.

**Table 5:** Observer usability assessment

	Yes		No	
	*n*	%	*n*	%
**Instructions for Use (IFU)**				
Did the study participant read the instructions for use (IFU)?^a^	859	95%	47	5%
If yes, were the instructions for use (IFU) read before the test?	701	82%	158	18%
Was the IFU referred to during the test process?	864	95%	42	5%
**Test Procedure**				
Did the study participant remove the contents of the test pack?	901	99%	5	1%
Was the study participant able to find the test tube packet?	905	100%	1	0%
Did the study participant remove the test tube from the packet?^a^	905	100%	1	0%
Did the study participant remove the cap from the test tube?^a^	896	99%	10	1%
Did the study participant place the test tube in the holder?^a^	848	94%	58	6%
Did the study participant have any difficulty with the test tube?	175	19%	731	81%
Was the study participant able to find the test stick packet?	903	100%	3	0%
Did the study participant remove the test stick from the packet?^a^	902	100%	4	0%
Did the study participant touch the flat pad?^a^	47	5%	859	95%
Did the study participant collect the sample correctly (1 × upper and lower swab)?^a^	754	83%	152	17%
Did the study participant place the test stick in the test tube correctly?^a^	889	98%	17	2%
**Procedure Check**				
Did the participant perform any steps out of the order?*	106	12%	800	88%
Did the participant miss any step and continue the process despite a missed or incorrect step?	46	5%	860	95%
Did the participant quit the process at any point?	1	0%	905	100%
Did the study participant wait 20 min before reading the results of the test?^a^	820	91%	86	9%

^a^Considered “critical” steps

Following the oral fluid sample collection, there were two categories of grouped procedure steps carried out as part of the test process: (1) performing the test procedure steps in order (88% did so correctly), and (2) waiting at least 20 min before interpreting the results (91% waited the correct time to interpret their results). Approximately 5% (*n* = 46) missed one or more steps but continued the self-test process despite this, and only one participant withdrew before the test was completed.

Participants’ self-test usability survey results, shown in Table 6, were compiled from participant responses. When asked about the instructions for use, 97% of participants (*n*=878) found them easy to follow. Regarding use of the device, 100% of responding participants indicated that they were confident with performing the test on their own, 99% (*n*=895) found the device easy to use, 98% of participants (*n*=882) indicated that they would use the test again and 99% (*n*=890) would recommend this test to a sexual partner or someone in their social network. In terms of preference in self-testing locations, 77% (*n*=697) indicated that they would prefer to do the test at home, while 23% (*n*=205) indicated that they would prefer to do the self-test at a clinic.

**Table 6:** Participant usability assessment

	Yes		No	
	*n*	%	*n*	%
**Instructions for Use (IFU)**				
Did you use the Instructions for Use (IFU) sheet to help you complete the test?	881	98%	22	2%
Were the Instructions for Use (IFU) easy to follow?	878	97%	24	3%
Were the pictures and illustrations in the Instruction for Use (IFU) helpful?	878	97%	24	3%
**Device Use**				
Was the Self-Test easy to use?	895	99%	7	1%
Are you confident you could perform this test on your own?	899	100%	3	0%
**Self-Test Experience**				
Would you use this test again if it were available to you?	882	98%	20	2%
Would you prefer to use this test at home (yes) or get tested at a clinic (no)?	697	77%	205	23%
Would you recommend this test to others? (e.g. a sexual partner, friend)	890	99%	12	1%

### Pre-made contrived test results interpretation

A total of 465 participants agreed to complete the pre-made contrived test result interpretation exercise. The six study sites were provided with 2 panels each, with each panel containing 5 blind-coded OraQuick® cartridges with the following pre-made contrived results:

1. Strong positive (control line with highly visible test line)
2. Weak positive (control line with weak but visible test line)
3. Negative (control line but no test line)
4. Invalid (no control line but a highly visible test line)
5. Invalid (no control line and no test line)

Participants were presented with the pre-made contrived results cartridges in singular random order and were asked to interpret the results for each. As shown in Table 7, the rate of correct interpretations varied across the different types of pre-made contrived results cartridges. For 3 of the 5 result types (strong positive, negative, and invalid with no control line and no test line), the rate of correct interpretations was ≥ 90%. For the “weak positive,” the correct interpretation mean was 70% (range 58–75%) which indicates that the majority of participants were able to see the line despite the weak intensity. The lowest rate of correct interpretations was obtained for the “invalid with a visible test line but no control line,” with a mean of 59% (range 49–80%), indicating that participants had some difficulties interpreting this type of invalid result. In follow-up analyses, most incorrect interpretations of the weak positive were recorded as negative, and for the invalid with no control line but a strongly visible test line most incorrect interpretations were reported as positive. There were no participant sociodemographic characteristics that were broadly associated with incorrect interpretations of the pre-made contrived results.

**Table 7:** Total pre-made contrived device result interpretations

	**Participant Interpretations (*n* = 465)**							
	Positive		Negative		Invalid		Do not know	
**Device Result**	*n*	%	*n*	%	*n*	%	*n*	%
Strong Positive	**418**	**90%**	4	1%	43	9%	0	0%
Weak Positive	**329**	**71%**	117	25%	15	3%	4	1%
Negative	6	1%	**453**	**97%**	4	1%	2	0%
Invalid with T Line	115	25%	53	11%	**273**	**59%**	24	5%
Invalid without T Line	0	0%	6	1%	**448**	**96%**	11	2%

## Discussion

This study was successful in demonstrating the excellent performance of the OraQuick® HIV self-test overall in meeting the expected results for the study objectives. The study outcome for negative percent agreement was comparable to specificity data from large-scale studies conducted in the US. The US data, summarized in the FDA Summary of Safety and Effectiveness also showed acceptable sensitivity [29], which led to FDA approval of the OraQuick® HIV Self-Test. Despite only encountering a single confirmed HIV positive in this study, the positive percent agreement and negative percent agreement with 4th generation HIV reference tests were 100%. The single positive was a previously undiagnosed individual who was living with HIV and was subsequently linked to care and treatment.

In terms of usability, there was a 97.6% concordance in the self-test results interpretation between the self-test study participants and the trained observers indicating that intended users of the OraQuick® HIV Self-Test can interpret their self-test results correctly compared to health care provider interpretations. Most discordant results between observer and participant were in the uncertainty (“do not know/not sure”) response category although the overall number of “do not know/not sure” results reported by participants was low (*n* = 9). A total of 20 participants (2.2% of the sample) interpreted their self-test results as invalid. Of these, 17 were also interpreted as invalid by the Observers. The rate of invalid results obtained by intended users of a self-test device is often an indication of ease of use and if some users may be experiencing difficulties in proper sample collection and/or performing the test procedures correctly. Previous Canadian studies of a blood-based HIV self-test had shown that rates of invalid results with self-testing by intended users could vary according to user experience with HIV testing and other contributing sociodemographic factors, with invalid rates of up to 20% reported [21, 30]. The low rate of invalid results observed in this study indicates that participants had few difficulties with performing the test procedures correctly on their own or in seeing a visible control line.

The pre-made contrived results interpretations for the 465 participants who volunteered for this portion of the study showed the majority were concordant with the expected results. There was high percent agreement, as expected, with 3 of the 5 pre-made contrived results: for the “strong positive,” “negative” and the “invalid, with no control or test line present.” However, results indicated that the “weak positive” and “invalid with a visible test line” can be somewhat more challenging to interpret correctly for some individuals. For the weak positive, it is to be expected that a test line with low intensity may not be consistently visualized. This outcome (70% correct interpretations) is similar to that reported by Majam et al. in their study of the performance of seven HIV-ST, including pre-made contrived results, in South Africa [28]. This does indicate a potential risk for a false negative interpretation based on visual acuity for discerning a test line of weak intensity, which is a limitation of all diagnostic devices that rely entirely on visual interpretation. For the invalid with no control line but a strongly visible test line (59% correct interpretations), this is unlikely a concern in real-life testing situations as this would rarely occur and would usually be an indication of a manufacturing flaw in a particular device, which had escaped the rigid quality control measures in place during manufacture. Although demographics such as visual impairment that was reported by 33% of participants in this study were not associated with higher rates of incorrect interpretations of pre-made contrived results, some mitigations may be considered to improve the instructions for interpreting the range of self-test results that users may experience, particularly with more challenging results such as a weak positive or invalid result with no control line but a visible test line. It would be important in the instructions to emphasize that the absence of a control line indicates an invalid result regardless of whether a test line is visible or not. An added instruction to conduct self-testing in a well-lit area would be beneficial for the user as well. Additional suggested modifications could include slight revisions to the package insert to provide clearer instructions and/or visual illustrations on the proper interpretation, as well as alerting the self-tester that even a faint line intensity is considered valid.

In general, participants in this study presented a broad diversity of sociodemographics that would be representative of the intended users of HIV self-tests in Canada. Participants expressed a high level of satisfaction with the HIV-ST experience as the vast majority of participants (97% to 99%) found the instructions for use easy to follow, found the self-test device easy to use, would use the OraQuick® HIV Self-Test again, and would recommend its use to a sexual partner or friend which aligns with other studies among a variety of populations in different global settings [10, 11, 14] and can lead to increases in HIV testing rates overall [6, 31, 32]. Moreover, results from a large-scale oral fluid self-test implementation program in the US indicated that a high percentage of persons in the program had never previously received testing for HIV or had not received testing within the previous year, especially in younger populations under 25 years of age [33]. In this current Canadian study, 47% of participants indicated that they were unaware of their HIV status or had never tested before, despite having access to free, licensed blood-based HIV self-tests. Oral fluid remains the most common sample substrate for HIV self-testing globally and oral fluid HIV self-tests are broadly utilized in various distribution strategies [32, 34, 35].

Overall, 17% of study participants were observed to have had some difficulties in the correct use of the swab for oral fluid collection, indicating that additional instructions might be necessary in some situations. Of interest, approximately 77% of participants indicated a preference for using the self-test in a home environment while 23% would prefer to use the test at a clinic or health care facility, suggesting that both assisted (supervised) and unassisted self-testing strategies could potentially be implemented to maximize “real world” access and uptake. Collectively, these outcomes indicate that this diverse, representative study population, with no prior experience with this oral fluid-based self-test, had a high degree of comfort and satisfaction with their self-testing experience.

Studies have shown that preferences for oral fluid or blood-based HIV self-testing devices vary depending on user perceptions of accuracy, sample type integrity, and ease of collection of the sample type [36–40]. However, self-test uptake overall could be enhanced by having both types of HIV self-tests licensed for use in Canada and offering users a choice of methods through the implementation of sustainable self-testing programs.

Despite the overall favourable outcomes from this study, there were some limitations: the study encountered only a single HIV-positive participant in the study population, including the 57% (*n* = 520) who reported at least one behaviour characteristic that would be considered “high risk”, therefore meaningful determination of the device sensitivity for accurate detection of HIV antibodies in the hands of intended users could not be measured. Survey responses from participants were voluntary and response rates varied across the individual questions, yielding variable denominators for determining outcomes for study participant sociodemographics and usability analyses. Lastly, a total of 73% of participants indicated they had a college or university education, which may be a potential bias and not truly representative of a broader and more educationally diverse intended user population for the self-test.

## Conclusions

We found the oral fluid-based OraQuick® HIV Self-Test to be highly accurate and usable by the diverse study participants. Since Canada has only a single licensed HIV self-test which is blood-based, having access to both blood-based and oral fluid-based HIV self-testing has the potential to substantially increase HIV testing rates. An approved, accessible oral fluid HIV self-test in Canada can offer an accurate, non-invasive, and easy-to-use alternative to facility-based HIV testing and to blood-based HIV self-testing for key/priority populations.

## Supplementary Information

Supplementary Material 1.

## Data Availability

The datasets used and/or analyzed during the current study are available from the corresponding author on reasonable request.

## Abbreviations

HIV: Human Immunodeficiency Virus
HIV-ST: HIV Self-Test
CM: Comparator Methods
CMIA: Chemiluminescent Microparticle Immunoassay
CONSORT: Consolidated Standards of Reporting Trails
IRB: Institutional Review Board
PPA: Positive Percent Agreement
NPA: Negative Percent Agreement
RDT: Rapid Diagnostic Test
REB: Research Ethics Board
WHO: World Health Organization
