# Supplementary material for: A prospective multi-site study to evaluate the performance and usability of an oral fluid-based HIV self-test in Canada
Source: BMC Public Health. 2025 Jan 11;25:125. doi: 10.1186/s12889-024-21228-8 (PMC11724549; doi:10.1186/s12889-024-21228-8)
Supplement: Supplementary file 1 — Supplementary Material 1. [file 12889_2024_21228_MOESM1_ESM.zip › OraQuick manuscript Appendix A clean final revised Dec 5.docx]

**A Prospective Multi-Site Study to Evaluate the Performance and Usability of an Oral Fluid-Based HIV Self-Test in Canada**

Appendix A

Inclusion criteria: participants considered eligible for inclusion were those who:

1. Are ≥18 years of age
2. Are able to speak/read/write English or French
3. Have presented for voluntary testing for HIV infection in the clinic or community-based setting
4. Are willing to participate in the study site’s standard of care HIV counseling and testing program and receive the study site’s standard of care test results
5. Are willing to be a participant in the study
6. Are able to provide informed consent i.e. understand and sign the informed consent form
7. Are able to complete the required testing on the allocated testing day
8. Are willing to provide the necessary oral fluid and venipuncture blood for use in the study protocol testing methods.
9. Are of unknown HIV status (last HIV negative test must be a minimum of 3 months prior)

Exclusion criteria included:

1. Are known HIV positive
2. Are on antiretroviral therapy (ART) or anti-HIV medications for the treatment of HIV, either as PrEP, PEP or experimental vaccine
3. Have any experience or have ever carried out rapid diagnostics tests for HIV or any other infectious disease on a patient.
4. Are investigator site employees or immediate family members of the sponsor or investigator site employee
5. Are currently participating in a concurrent trial of HIV self-tests
6. Are a practicing medical healthcare professional (doctor, nurse, or HIV counselor that performs HIV testing with Rapid Tests)
7. Any condition which, in the opinion of the Observer, would make the participant unsuitable or unsafe for enrolment or could interfere with the completion of the assessment and questionnaire, etc. or bias the outcome, e.g. being unable to see/read by forgetting to bring reading glasses, being intoxicated, acute sickness, visibly distressed.
