# Supplementary material for: A prospective multi-site study to evaluate the performance and usability of an oral fluid-based HIV self-test in Canada
Source: BMC Public Health. 2025 Jan 11;25:125. doi: 10.1186/s12889-024-21228-8 (PMC11724549; doi:10.1186/s12889-024-21228-8)
Supplement: Supplementary file 1 — Supplementary Material 1. [file 12889_2024_21228_MOESM1_ESM.zip › OraQuick manuscript Appendix B_final_clean_revised Dec 5.docx]

**A Prospective Multi-Site Study to Evaluate the Performance and Usability of an Oral Fluid-Based HIV Self-Test in Canada**

**Appendix B: Study Exclusions**

**Table 1: Exclusions From All Analyses (n=40)**

| **Number of Participants** | **Reason for Exclusion** |
| --- | --- |
| 7 | Met one or more exclusion criteria |
| 21 | No self Test result |
| 10 | No laboratory result |
| 2 | Protocol violation/data entry error |

**Table 2 – Pre-made contrived results exercise exclusions (n=10)**

| **Number of Participants** | **Reason for Exclusion** | **Notes** |
| --- | --- | --- |
| 10 | Duplicate mock result panel letter entered in the database; unable to verify which panel was used. | Included in performance analysis, but excluded from mock device exercise |
