# Supplementary material for: A prospective multi-site study to evaluate the performance and usability of an oral fluid-based HIV self-test in Canada
Source: BMC Public Health. 2025 Jan 11;25:125. doi: 10.1186/s12889-024-21228-8 (PMC11724549; doi:10.1186/s12889-024-21228-8)
Supplement: Supplementary file 1 — Supplementary Material 1. [file 12889_2024_21228_MOESM1_ESM.zip › OraQuick Manuscript Appendix C_clean_ revised Dec 5.docx]

**A Prospective Multi-Site Study to Evaluate the Performance and Usability of an Oral Fluid-Based HIV Self-Test in Canada**

**Appendix C: Study Survey Tools**

1. **Enrolment Questionnaire**

**********************************************************************************

**PARTICIPANT ENROLLMENT Questionnaire**

**This section is to be completed by research staff using verbal responses from participants**

**********************************************************************************

**You are now completing the ELIGIBILITY section of the questionnaire.**

1. **Participant has read and consented to take part in this research study:** (select only one)

Yes  No (If No, cannot proceed)

1. **Date of consent:** (dd/mmm/yyyy) [validated entry]
2. **Approximate time of consent:** (hh:mm AM/PM) [validated entry]
3. **Site ID#:** (assigned) [text box]
4. **Inclusion criteria:**

Participant considered eligible for inclusion are those who meet all of the following:

| Number | Inclusion Criteria | YES | NO |
| --- | --- | --- | --- |
| **5.1** | Are you 18 years of age or older? |  |  |
| **5.2** | Are able to speak / read / write English or French? |  |  |
| **5.3** | Are you of unknown HIV status **OR** Has your last HIV negative test been more than three (3) months ago (prior to the study visit) |  |  |
| **5.4** | Have presented for voluntary testing for HIV infection in the clinic or community based setting |  |  |
| **5.5** | Are willing to participate in the study site’s standard of care HIV counselling and testing program and receive the study site’s standard of care test results |  |  |
| **5.6** | Are willing to be a participant in the research study |  |  |
| **5.7** | Are willing to use undergo testing with the OraQuick ADVANCE ® HIV-1/2 Rapid Antibody Test |  |  |
| **5.8** | Are able to complete the required testing on the allocated testing day |  |  |
| **5.9** | Are willing to provide the necessary oral fluid and venipuncture blood for use in the study protocol testing methods |  |  |

1. **Exclusion criteria:**

Participant considered to be ineligible for participation are those who meet *any o*f the following:

| Number | Exclusion Criteria | NO | Yes |
| --- | --- | --- | --- |
| **6.1** | Do not meet all the inclusion criteria |  |  |
| **6.2** | Are known HIV positive |  |  |
| **6.3** | Are on antiretroviral therapy (ART) or anti-HIV medications for the treatment of HIV, either as pre-exposure prophylaxis (PrEP), post-exposure prophylaxis (PEP) or experimental HIV vaccine?  **6.3.1** If Yes, please specify: [text box] |  |  |
| **6.4** | Have any experience or have ever conducted a rapid diagnostic self-test for HIV (including OraQuick HIV Self-Test), HCV (hepatitis C virus) or any other sexually transmitted blood-borne infection (STBBI)?  **6.4.1** If Yes, please specify: [text box] |  |  |
| **6.5** | Are currently participating in a concurrent trial of HIV self-tests? |  |  |
| **6.6** | Are investigator site employees or immediate family members of sponsor or investigator site employee? |  |  |
| **6.7** | Are a practicing medical healthcare professional (doctor, nurse or HIV counsellor that performs HIV testing with Rapid Tests)? |  |  |
| **6.8** | Any condition which, in the opinion of the Observer, would make the participant unsuitable or unsafe for enrolment or could interfere with the completion of the assessment and questionnaire etc. or bias the outcome, e.g. being unable to see / read by forgetting to bring reading glasses, being intoxicated, acute sickness, visibly distressed. ***Please specify*:** [text box] |  |  |

1. **Results of Enrollment:**

**REMINDER: All exclusion criteria must be answered NO for patient to qualify for this study.**

Enrolled

Excluded

Withdrawn, please specify reason: [text box]

1. **Eligibility Criteria Reviewed By:** (Observer ID#/Initials)[text box]

**You are now completing the Enrollment section of the questionnaire.**

**This includes demographics, social and medical history.**

1. **Enrolled, Participant ID:** [validated entry]

| **Study Name** | **Province** | **Site Code** | **Participant Code** |
| --- | --- | --- | --- |
| (2 Letters) | (2 Letters | (2 Digits) | (3 Digits) |

1. **Was a venous blood draw collected from the participant** (for confirmatory testing)? (select only one)  Yes  No

**10.1 If No**, please explain primary reason for premature exit: [text box]

**10.2 If Yes,** please provide:

**10.2.1 Date of collection:** (dd/mmm/yyyy) [validated entry]

**10.2.2 Time of collection:** (hh:mm AM/PM) [validated entry]

**10.2.3 Sample collected by:** (select only one):  Phlebotomist  Nurse or other Health Care Provider  Other, please specify: [text box]

1. **What is your current age?** (select only one)

18-25

26-35

36-45

46-55

>55

Prefer not to answer

1. **What is your current gender identity?** (select only one)

Woman

Man

Trans woman

Trans man

Non-Binary

Genderqueer

I prefer to describe myself as (please specify): [text box]

Prefer not to answer

1. **The sex you were assigned at birth (meaning on my original birth certificate) was?** (select only one)

Male

Female

Prefer not to answer

1. **How do you identify?** (select all that apply)

Indigenous (e.g. First Nations, Inuit, Métis) in what is sometimes referred to as Canada

**14.1** **I am:** (if Indigenous selected)

First Nations

Métis

Inuk (Inuit)

Prefer to describe: [text box]

Prefer not to answer

**14.2** **Are you Two-Spirit?** (if Indigenous selected)

Yes

No

Prefer not to answer

Indigenous outside of what is sometimes referred to as Canada

Latino, Latina, or Latinx (e.g. Mexican, Central/South American

South Asian (e.g. East Indian, Pakistani, Sri Lankan, Punjabi, Bangladeshi)

Arab, West Asian, North African (e.g. Armenian, Iranian, Lebanese, Afghani, Egyptian)

East Asian (e.g. Chinese, Japanese, Vietnamese, Cambodian, Indonesian, Filipino, Korean, Laotian)

African (e.g. Somalian, Congolese, Namibian)

Caribbean (e.g. Jamaican, Haitian)

Black

White

Other (please specify): [text box]

Prefer not to answer

1. **How would you describe your sexuality?** (select all that apply)

Gay

Lesbian

Bisexual

Queer

Straight

Questioning

Asexual

Pansexual

Heteroflexible (“mostly straight”)

I prefer to describe myself as (please specify): [text box]

Prefer not to answer

1. **What is your highest education level achieved?** (select only one)

No formal education (none)

Primary

Secondary (High School)

College or non-university (e.g. trade school)

University (bachelors) or higher (e.g. masters, PhD)

Prefer not to answer

1. **What is your current employment status?** (select all that apply)

Employed

Student

Retired

Unemployed

Prefer not to answer

1. **Medical Health** - What are your self-reported medical conditions? (select all that apply)

Diabetes Mellitus

**18.1** If selected, provide approximate date of diagnosis: [text box]

Hypertension

**18.2** If selected, provide approximate date of diagnosis: [text box]

Visual Impairment

**18.3** If selected, provide approximate date of diagnosis: [text box]

Existing/Recent Sexually transmitted diseases (example: Chlamydia, Gonorrhea, etc.)

**18.4** If selected, please specify and provide approximate date of diagnosis: [text box]

Other, please specify and provide approximate date of diagnosis for each: [text box]

1. **Are you pregnant?**

Yes

No

Not applicable

1. **What risk categories do you identify with?** (select all that apply)

Unprotected sex with men

Unprotected sex with women

Multiple sexual partners

Had a sexually transmitted infection (STI) (e.g. syphilis, herpes, chlamydia, gonorrhea and

bacterial vaginosis) or diagnosed with Hepatitis C

Injection drug user

Engage in sex work

Born to an HIV positive mother

Sexual partner is HIV positive

Sexual partner is a bisexual male

Received a blood or blood product transfusion or organ transplant in a country that does not

screen blood products for HIV

Other, please specify: [text box]

1. **Do you have experience with HIV testing?** (select only one) Yes No

**21.1** **If YES, how have you tested for HIV in the past?** (select all that apply)

I had blood taken at a lab/office (e.g. blood drawn from the arm)

I had a point-of-care test (e.g. fingerstick test)

I used an HIV Self-Test* ***(if self-test used, then apply exclusion criteria)***

I performed a point-of-care test on someone ***(if performed POC test, then apply exclusion criteria)***

1. **What is your self-reported HIV status?**

Unknown status/never been tested

Negative status

**22.1 If NEGATIVE, what was the approximate date of your last HIV test?** (mmm-yyyy) [validated] ***(if negative test date is less than 3 months, then apply EXCLUSION CRITERIA)***

**22.2 If NEGATIVE, obtain location and type of test used:** [text box]

Positive status*

**22.3 If POSITIVE, obtain the date, location and type of test used, if known:** [text box] ***(if HIV positive, then apply EXCLUSION CRITERIA)***

1. **Have you ever received any anti-HIV medicines?** (select only one): Yes No
   1. **If YES, what type of anti-HIV medicines have you used:** (select all that apply)

Post-Exposure prophylaxis (PEP), a 4-week course of medications that you can take if you are HIV negative and think you have been recently exposed to HIV

Pre-exposure prophylaxis (PrEP), a daily medication to help prevent HIV infection

Treatment for HIV, medicines taken regularly to manage your HIV infection

1. **Are you currently taking any of the following medications**? (select all that apply)

Herbal or dietary supplements (please specify: [text box])

Any medication used to treat or prevent HIV (ARV and/or PrEP)* ***(if taking these treatments, apply EXCLUSION CRITERA)***

**24.1** **If YES, please record below:**

| **Medication Name**  (Brand or Generic Name) | **Dosage Form** | **Frequency*** | **Start Date**  (mmm/yyyy) | **Ongoing?**  (Yes or No) | **End Date**  (if applicable,  mmm/yyyy) |
| --- | --- | --- | --- | --- | --- |
|  |  |  |  |  |  |
|  |  |  |  |  |  |
| *Frequency Codes: QD (every day), OTH (other, specify) | | | | | |

1. **What is your preferred (primary) official language?**

English

French

1. **Can you read and understand what you have read in English?** (select only one) ☐ Yes ☐ No
2. **Can you read and understand what you have read in French?** (select only one) ☐ Yes ☐ No
3. **Do you have any form of reading/writing impairment?**

☐ No

Yes

**28.1 If YES,** please specify: [text box]

1. **Ocular Health** - Do you have any form of visual impairment?

No

Yes

**29.1 If YES,** do you:

Wear corrective lenses e.g. glasses or contact lenses?

Have any condition that specifically affects vision, besides the need of corrective lenses, e.g. macular degeneration/ cataract/ glaucoma?

Other, please specify: [text box]

1. **Oral Health** - What are your self-reported oral conditions? (Select all that apply)

Cavities and/or fillings

Braces

Periodontal disease and/or bleeding gums

Oral fixtures; full or partial dentures

Other, please specify: [text box]

None

1. **What was your specific last use of oral products?**

Food and/or beverage consumption (gum or mints included)

Tooth paste

Mouthwash or oral rinse

Smoking (including vaping products) or smokeless tobacco (including chewing tobacco)

**31.1 How long ago did you use this oral product?**

Less than 15 mins

15 mins – 1 hour

1hr – 24 hours

More than 24 hours

1. **Date data collected:** (dd/mmm/yyyy) [validated entry]
2. **Time data collected:** (hh:mm AM/PM) [validated entry]
3. **Data entered by:** (Observer ID#/ Initials) [text box]
4. **Participant's OraQuick^®^ HIV Self-Test Result Record Form**

**********************************************************************************

**Participant's OraQuick® HIV Self-Test Result Record Form**

**To be completed by research staff using observations and verbal responses from participants.**

**********************************************************************************

**The observer will complete this form based on the participant's responses to their own OraQuick^®^ Self-Test result**

Observer: *“I am not sure what you think your test result is. According to the product instructions, can you please tell me what you think the result of your test is?”*

**Note:** Any ambiguous verbal answer by the participant will have one attempt made to clarify it.

1. **What is the Participant's interpretation of their OraQuick® result?** (select only one)

Negative / Do not have HIV

Positive / May have HIV

Invalid/ Test did not work

Don’t know/ Not sure

Refused or ambiguous answer

Could not complete OraQuick^®^ HIV self-test

1. **Additional Interpretation Comments/reason(s) for incomplete Self-Test:** [Text box]
2. **Is the test impossible to read?**(select only one)

☐ No

☐ Yes

**3.1** If Yes, please explain why: [Text box]

1. **Date data collected:** (dd/mmm/yyyy) [validated entry]
2. **Time data collected:** (hh:mm AM/PM) [validated entry]
3. **Data entered by:** (Observer ID#/ Initials) [text box]
4. **Participant's Mock OraQuick^®^ Result Interpretation Record Form**

   **********************************************************************************

**Participant's OraQuick® HIV Self-Test Result Record Form**

**To be completed by research staff using observations and verbal responses from participants.**

******************************************************************************

**The observer will complete this form for recording the Mock OraQuick^®^ HIV Self-Test result interpretation from the participant.**

1. **Was the participant asked to do a Mock Test?** (select only one)

Yes

No

1. **Did the participant complete the Mock Test?** (select only one)

Yes

No

**8.1** **If NO**, please explain why: [text box]

**The mock tests should be given to participants in a RANDOM order. Please pass the test kits to the randomized order indicated immediately below. (select all that apply)
Please note order in which this survey asks the questions will always remain the same, and you may have to scroll up/down to respond to the corresponding question.**

Strong Positive

Weak Positive

Negative

Invalid – no control line present, test line present

Invalid – no control line present, no test line present

1. **True Mock Test Result: Strong Positive**

**3.1 What is the participant's interpretation of the Mock Test Result?** (select only one)

Positive

Negative

Invalid

Do not know/Not sure/Quit Process

- 1. **Does the participant’s interpretation match the true mock result?** (select only one)

Yes

No

1. **True Mock Test Result: Weak Positive**

**4.1 What is the participant's interpretation of the Mock Test Result?** (select only one)

Positive

Negative

Invalid

Do not know/Not sure/Quit Process

**4.2 Does the participant’s interpretation match the true mock result?** (select only one)

Yes

No

1. **True Mock Test Result: Negative**
   1. **What is the participant's interpretation of the Mock Test Result?** (select only one)

Positive

Negative

Invalid

Do not know/Not sure/Quit Process

- 1. **Does the participant’s interpretation match the true mock result?** (select only one)

Yes

No

1. **True Mock Test Result: Invalid - no control line present, test line present**
   1. **What is the participant's interpretation of the Mock Test Result?** (select only one)

Positive

Negative

Invalid

Do not know/Not sure/Quit Process

- 1. **Does the participant’s interpretation match the true mock result?** (select only one)

Yes

No

1. **True Mock Test Result: Invalid - no control line present, no test line present**
   1. **What is the participant's interpretation of the Mock Test Result?** (select only one)

Positive

Negative

Invalid

Do not know/Not sure/Quit Process

- 1. **Does the participant’s interpretation match the true mock result?** (select only one)

Yes

No

1. **Comments on mock test interpretation** *(optional, please provide details)***:** [text box]
2. **Date data collected:** (dd/mmm/yyyy) [validated entry]
3. **Time data collected:** (hh:mm AM/PM) [validated entry]
4. **Data entered by:** (Observer ID#/ Initials) [text box]
5. **Observer's Data Collection Form - Observational Ratings of Study Participant-Self Test Performance**

**********************************************************************************

**Observer's Data Collection Form**

**To be completed by research staff using observations of participants.**

**********************************************************************************

**PART A: Observer’s report on their observation of the participant.**

1. **What language instruction of the IFU did the participant use?** (select only one)

English French Both

1. **What time** did the participant **start the test**? Record time when the participant opens the test pouch: (hh:mm AM/PM) [validated]
2. **Did the study participant read the instructions for Use (IFU)?** (select only one)

Yes No

**3.1 If YES,** were the Instructions for Use (IFU) read before the test?

Yes No

1. **Was the IFU referred to during the test process?**

Yes No

1. Did the study participant to **remove the contents of the test pack?**

Yes No

1. Was the study participant able to **find the test tube packet?**

Yes No

**6.1 If NO**, describe what seemed to be the problem (e.g. could not find tear point, weakness, confusion)? [text box]

1. Did the study participant **remove the test tube from the packet**?

Yes No

**7.1 If NO**, describe what seemed to be the problem (e.g. could not find tear point, weakness, confusion)? [text box]

1. Did the study participant **remove the cap from the test tube?**

Yes No

**8.1 If NO,** describe what seemed to be the problem? [text box]

1. **Did the study participant place the test tube in the holder?**

Yes No

**9.1 If NO,** describe what seemed to be the problem? [text box]

1. **Did the study participant have any difficulty with the test tube?**

Yes No

**10.1 If YES,** describe what seemed to be the problem? [text box]

1. Was the study participant able to **find the test stick** packet?

Yes No

**11.1 If NO,** describe what seemed to be the problem? [text box]

1. Did the study participant **remove the test stick** from the packet?

Yes No

**12.1 If NO**, describe what seemed to be the problem? [text box]

1. Did the study participant **touch the flat pad**?

Yes No

**13.1 If NO**, describe what seemed to be the problem? [text box]

1. Did the study participant **collect the sample correctly** (1x upper and lower swab)?

Yes No

**14.1 If NO,** describe what seemed to be the problem? [text box]

1. Did the study participant **place the test stick in the test tube correctly**? i.e. put the flat pad of the test stick into the tube until it touched the bottom

Yes No

**15.1 If NO**, describe what seemed to be the problem? [text box]

1. What **time** did the **participant conclude/ complete the test?** (hh:mm AM/PM) [validated]
2. Did the participant perform **any steps out of the order**? For example, did they place the test stick in the tube before completing the oral swab?

Yes No

**17.1 If YES**, please explain? [text box]

1. Did the participant **miss any step and continued the process** despite a missed or incorrect step?

Yes No

**18.1 If YES,** please explain? [text box]

1. Did the participant **quit the process** at any point?

Yes No

**19.1 If YES**, please explain? [text box]

1. **What was the participant’s apparent level of stress?** (select all that apply)

Calm

Appears anxious

Verbally communicates distress

Staff intervention required

Any other observer comments: [text box]

Additional notes (Was there significant hesitation or indecision at specific steps or overall? Did they say anything; did they ask any questions of the interviewer during the process? Which steps were skipped or modified?): [text box]

1. Did the study participant wait 20 minutes before reading the results of the test?

Yes No

1. **End time** - What **time** did the **participant read the test**: (hh:mm AM/PM) [validated]

**PART B: Observer’s Interpretation of the Participant’s OraQuick^®^ HIV Self-Test Result**

1. **Is the control (“C”) line present?**

Yes No

1. **Is the test (“T”) line present?**

Yes No

1. **Is the test impossible to read?**

Yes No

**25.1 If YES**, please explain? [text box]

1. **Observer's interpretation** of OraQuick^®^ result?

Negative / Do not have HIV

Positive / May have HIV

Invalid / Test did not work

Don’t know / Not sure

Participants could not complete OraQuick^®^ HIV self-test

1. **Date data collected:** (dd/mmm/yyyy) [validated entry]
2. **Time data collected:** (hh:mm AM/PM) [validated entry]
3. **Data entered by:** (Observer ID#/ Initials) [text box]
4. **Self-Test Questionnaire with Participant’s Responses**

******************************************************************************

**Self-Test Questionnaire**

**This section is to be completed by research staff using verbal responses from participants**

******************************************************************************

**The observer will ask the following questions to the participant and complete the questionnaire with the participant's answers.**

1. **Did you use the Instructions for Use (IFU) sheet to help you complete the test?**

Yes No

**1.1 If NO,** please explain: [text box]

1. **Were the Instructions for Use (IFU)** **easy to follow**?

Yes No

**2.1 If NO,** please explain: [text box]

1. **Were the pictures and illustrations in the Instructions for Use (IFU) helpful?**

Yes No

**3.1 If NO,** please explain: [text box]

1. Please **look at the Instructions for Use** (IFU) in front of you, and **show me any part which you did not read:** (List the section) [text box]
2. Please **look at the Instructions for Use** (IFU) and **show me which part was hard to understand:** (List the section) [text box]
3. **Was the easy to use?**

Yes No

**6.1 If NO,** please explain the steps that were difficult or confusing: [text box]

1. **Are you confident you could perform this test on your own?**

Yes No

**7.1 If NO,** please explain why you were not: [text box]

1. **How old** do you have **to** be **use this product?**

At least 17 years old

18 years or older

Older than 25 but less than 40 years

40 years and older

1. **Can you use this product if you are undergoing HIV treatment such as PrEP (pre-exposure prophylaxis) or PEP (post-exposure prophylaxis)?**

Yes

No

1. **How long after you place the device in the vial can you read the results?**

Less than 20 minutes

Between 20 to 40 minutes

More than 40 minutes

More than 60 minutes

1. **How long** must you **wait to use** the product **after consuming food or beverages?**

At least 10 minutes

At least 15 minutes

At least 20 minutes

At least 30 minutes

1. **How long** must you **wait to use** the product **after using mouth cleaning products**?

At least 10 minutes

At least 15 minutes

At least 20 minutes

At least 30 minutes

1. **What would you do next if your test results were negative?** [text box]
2. **What would you do next if your test results were positive?** [text box]
3. **What would you do next if your test results were invalid?** [text box]
4. **What should you do if you are not sure of your result?** [text box]
5. Would you **use this test again** if it were available to you?

Yes No

**17.1 If NO,** please explain why: [text box]

1. Would you **prefer to use this test at home or get tested at a clinic**?

Home Clinic

**18.1** Please explain your choice: [text box]

1. Would you **recommend** this test to others? E.g. a sexual partner/friend?

Yes No

**19.1 If NO,** please explain why: [text box]

1. Do you have suggestions on how to make this **product easier to use**? [text box]
2. Do you have suggestions on how to make the **IFU easier to follow**? [text box]
3. Do you have any **other comments about the test or your experience with self-testing**? [text box]
4. **Date data collected:** (dd/mmm/yyyy) [validated entry]
5. **Time data collected:** (hh:mm AM/PM) [validated entry]
6. **Data entered by:** (Observer ID#/ Initials) [text box]
7. **Visit 1 Completion Form**

******************************************************************************

**Visit 1 Completion Form**

**This section to be completed by research staff based on observation of study procedures.**

******************************************************************************

**REMINDER: To be completed for all participants.**

1. **Site ID#:** (assigned) [text box]
2. **Participant ID#:** [validated entry]

| **Study Name** | **Province** | **Site Code** | **Participant Code** |
| --- | --- | --- | --- |
| (2 Letters) | (2 Letters | (2 Digits) | (3 Digits) |

1. **Did the participant complete Visit 1 as planned?**: (select only one) Yes No

**3.1 If NO, indicate primary reason for premature exit**:

Participant withdrew consent

Inability to collect a venous whole blood sample from participant

Participant experienced an adverse event*

**REMINDER: Complete and append the Adverse Event Form**

Other, specify: [text box]

1. **Date data collected:** (dd/mmm/yyyy) [validated]
2. **Time data collected:** (hh:mm AM/PM) [validated]
3. **Data entered by:** (Observer ID#/ Initials) [text box]
